# Development and Characterization of a Macaque Model of Focal Internal Capsular Infarcts

**DOI:** 10.1371/journal.pone.0154752

**Published:** 2016-05-05

**Authors:** Yumi Murata, Noriyuki Higo

**Affiliations:** Human Informatics Research Institute, National Institute of Advanced Industrial Science and Technology (AIST), Umezono, Tsukuba, Ibaraki, Japan; University of Münster, GERMANY

## Abstract

Several studies have used macaque monkeys with lesions induced in the primary motor cortex (M1) to investigate the recovery of motor function after brain damage. However, in human stroke patients, the severity and outcome of motor impairments depend on the degree of damage to the white matter, especially that in the posterior internal capsule, which carries corticospinal tracts. To bridge the gap between results obtained in M1-lesioned macaques and the development of clinical intervention strategies, we established a method of inducing focal infarcts at the posterior internal capsule of macaque monkeys by injecting endothelin-1 (ET-1), a vasoconstrictor peptide. The infarcts expanded between 3 days and 1 week after ET-1 injection. The infarct volume in each macaque was negatively correlated with precision grip performance 3 days and 1 week after injection, suggesting that the degree of infarct expansion may have been a cause of the impairment in hand movements during the early stage. Although the infarct volume decreased and gross movement improved, impairment of dexterous hand movements remained until the end of the behavioral and imaging experiments at 3 months after ET-1 injection. A decrease in the abundance of large neurons in M1, from which the descending motor tracts originate, was associated with this later-stage impairment. The present model is useful not only for studying neurological changes underlying deficits and recovery but also for testing therapeutic interventions after white matter infarcts in primates.

## Introduction

Several studies, including ours, have used macaque monkeys with lesions induced in the primary motor cortex (M1) to investigate the recovery of motor function after brain damage [1–8]. M1 lesions specifically impair motor function, leaving sensory and cognitive functions intact; they are therefore suitable for investigating changes in neural structure and function that are associated with deficits and recovery of motor behavior. However, in human stroke patients, the severity and outcome of motor impairments depend on the degree of damage to the white matter, especially that in the posterior internal capsule, which carries corticospinal tracts [9–11]. Thus, to bridge the gap between the results obtained in M1-lesioned macaques and the development of clinical intervention strategies, it is important to establish a non-human primate model of focal stroke at the posterior internal capsule.

Several models of focal stroke at the internal capsule have been established in the rat [12–15] and miniature pig [16]. Recently, an internal capsule stroke model was also produced in the common marmoset [17], a small non-human primate species used widely in basic and clinical research because of its ease of handling and breeding. These models are useful for studying the neurological changes underlying behavioral deficits and recovery after stroke in the white matter. However, the macaque has an advantage over other experimental animals because it is more compatible with humans with regard to both molecular and anatomical features of the motor cortex and corticospinal tract than other species used experimentally, including squirrel monkeys and marmosets, thus making the findings more applicable to human patients [18–21]. Here, we established a method of inducing focal infarcts at the posterior internal capsule by injecting endothelin-1 (ET-1), a vasoconstrictor peptide [22], and investigated behavioral and histological changes after injection.

## Materials and Methods

### Animals

Ten Japanese macaques (*Macaca fuscata*, eight males and two females, 4.2 to 8.8 kg, > 4 years in age) were randomly assigned to one of five survival periods, namely 4 days, 2 weeks, 3 weeks, 1 month, and 3 months, after ET-1 injection. Two macaques (two males, 4.2 and 6.3 kg) died within few hours after ET-1 injection due to respiratory failure, and the remaining eight macaques were used for the present analysis (Table 1). Brain tissues were also obtained from six intact Japanese macaques (four males and two females, 3.1 to 8.9 kg) as controls for histological analysis. The macaques were either purchased from a local provider (Kawahara Bird-Animal Trading Co. Ltd., Tokyo, Japan) or bred in the Primate Research Institute of Kyoto University. No statistical methods were used to pre-determine sample sizes. We attempted to minimize the number of monkeys used on the basis of ethical considerations and data similarity; our sample sizes are similar to those reported in previous publications by our group and others. Naive macaques without any history of experimentation were used.

**Table 1:** List of the endthelin-1 (ET-1) injected monkeys studied.

	Sex	Weight, kg	Infarct volume at 1 day after injection, mm^3^ (% within the internal capsule)	Survival period after injection
**Mk-Ce**	male	5.6	597.0 (82.4)	4 days
**Mk-Ta**	male	8.2	937.5 (72.2)	4 days
**Mk-Um**	female	6.0	361.5 (81.7)	2 weeks
**Mk-Ma**	male	5.2	801.0 (73.2)	3 weeks
**Mk-Du**	male	6.0	427.5 (85.6)	1 month
**Mk-Ku**	male	8.8	436.5 (82.1)	3 months
**Mk-Mu**	female	4.6	546.0 (83.2)	3 months
**Mk-Sa**	male	5.2	321.0 (91.6)	3 months

The animal use protocol, including the mortality aspects, was reviewed and approved by the Institutional Animal Care and Use Committee of the National Institute of Advanced Industrial Science and Technology (AIST), Japan (#2012–049), conformed to the NIH Guidelines for the Care and Use of Laboratory Animals and ARRIVE guidelines (S1 ARRIVE Guidelines Checklist). Our procedure also followed the recommendations of the Weatherall report [23]. The macaques were housed in adjoining individual primate cages allowing social interactions under controlled conditions of humidity, temperature, and light; they were monitored daily by the researchers and animal care staff to ensure their health and welfare. The housing area was maintained on a 12-h light–12-h dark cycle, and all of the experiments were conducted during the light cycle. A commercial primate diet and fresh fruit and vegetables were provided daily, and water was provided in a drinking bottle and freshened daily. Environmental enrichment consisted of commercial toys and a platform placed at the middle of the cage wall which enabled the macaques to freely move up and down. All surgery was performed under sodium pentobarbital anesthesia, and all efforts were made to minimize suffering. Endpoint criteria, as defined by the study protocol, were used to determine when animals should be humanely euthanized; macaques found in a moribund condition and those showing severe pain and/or enduring signs of severe distress should be humanely euthanized. Two macaques died within few hours after ET-1 injection as described above. They died before awakening from anesthesia without showing any signs of pain or distress. No macaque met the humane endpoint criteria in the present study.

### ET-1 Injection

We first determined the preferred hand of macaques by recording the hand that was used for reaching and grasping the target object, as in our previous studies [1, 2]. We then injected ET-1 into the posterior internal capsule of the hemisphere contralateral to the preferred hand. The location of the posterior internal capsule where the fibers from the M1 hand area descend was identified by using anatomical structures such as the central sulcus, thalamus, caudate nucleus, and putamen on the basis of data from anatomical tracer studies [24, 25]. The anatomical structure of each macaque’s brain was visualized on magnetic resonance images (MRI) obtained by using a 3.0-T MRI system (3T Signa LX; General Electric Medical Systems, Milwaukee, WI). Before the scan, the animals were anesthetized with medetomidine (0.05 mg/kg), midazolam (0.3 mg/kg), and ketamine (4 mg/kg) and fixed into a magnet-free stereotactic frame. The imaging protocols consisted of a T1-weighted spoiled gradient-echo sequence, repetition time (TR)/echo time (TE), 50.0/9.0 ms; number of excitations (NEX), 1; flip angle, 45°; field of view, 120 mm × 120 mm; matrix, 256 × 192; slice thickness, 1.2 mm, and a T2-weighted fast spin echo sequence, TR/TE, 5000/102 ms; NEX, 4; flip angle, 90°; field of view, 120 mm × 120 mm; matrix, 256 × 256; slice thickness, 1.5 mm,. After incision of the head skin, the skull was exposed and an MR-compatible chamber was attached to the skull under sterile conditions and pentobarbital anesthesia (25 mg/kg). A craniotomy was made over the internal capsule, and ET-1 (1.5 μg/μL, 4198-v, Peptide Institute, Inc., Osaka, Japan) was then injected via a microsyringe. A total of 15 injections were performed at three injection sites, separated by 1.5 mm in a dorsoventral direction, in each of five rostral–caudal tracks, which were also separated by 1.5 mm. The injection sites were centered at the identified stereotaxic coordinates for the posterior internal capsule where the motor tracts from the hand area of M1 descend (*i*.*e*., the white matter lateral to the caudate nucleus and medial to the putamen in the coronal section 3 mm caudal to the rostrocaudal level where the rostral end of the central sulcus was seen). The mean stereotaxic coordinates of the center of the injection sites were 16.6 ± 3.7 (SD) mm rostral, 11.2 ±1.8 mm lateral, and 14.3 ± 2.0 mm below the dura. At each injection site, 8 μL ET-1 was injected at a rate of 2 μL per min. After injection, the needle was retained for 10 min to allow the solution to disperse and minimize backflow through the needle track.

### Behavioral Assessment

Performance of hand movements before and after the ET-1 injections was evaluated daily by means of a small-object retrieval task closely resembling that used in our previous studies [1, 2]. In the test session with this task, the macaques retrieved a small piece of sweet potato (7 × 7 × 7 mm in size) attached to a needle tip in a cylindrical tube (20 mm in diameter) for 20 trials. The cylindrical tube was located at shoulder height and at a sagittal distance of 150 mm from the cage. The macaque’s movements were recorded by four video cameras (one HDC-TM350 camera, Panasonic, Osaka, Japan; three WAT-902H Ultimate cameras, Watec, Tsuruoka, Japan) installed around the task apparatus. We calculated the percentage of retrieval, *i*.*e*., grasping and eating the food piece/pellet without dropping it, and that of precision grip, defined as grasping the food piece between the pads of the tips of the index finger and thumb without using other digits or the palm.

### Infarct Size Assessment and Histology

**Fig 1:**
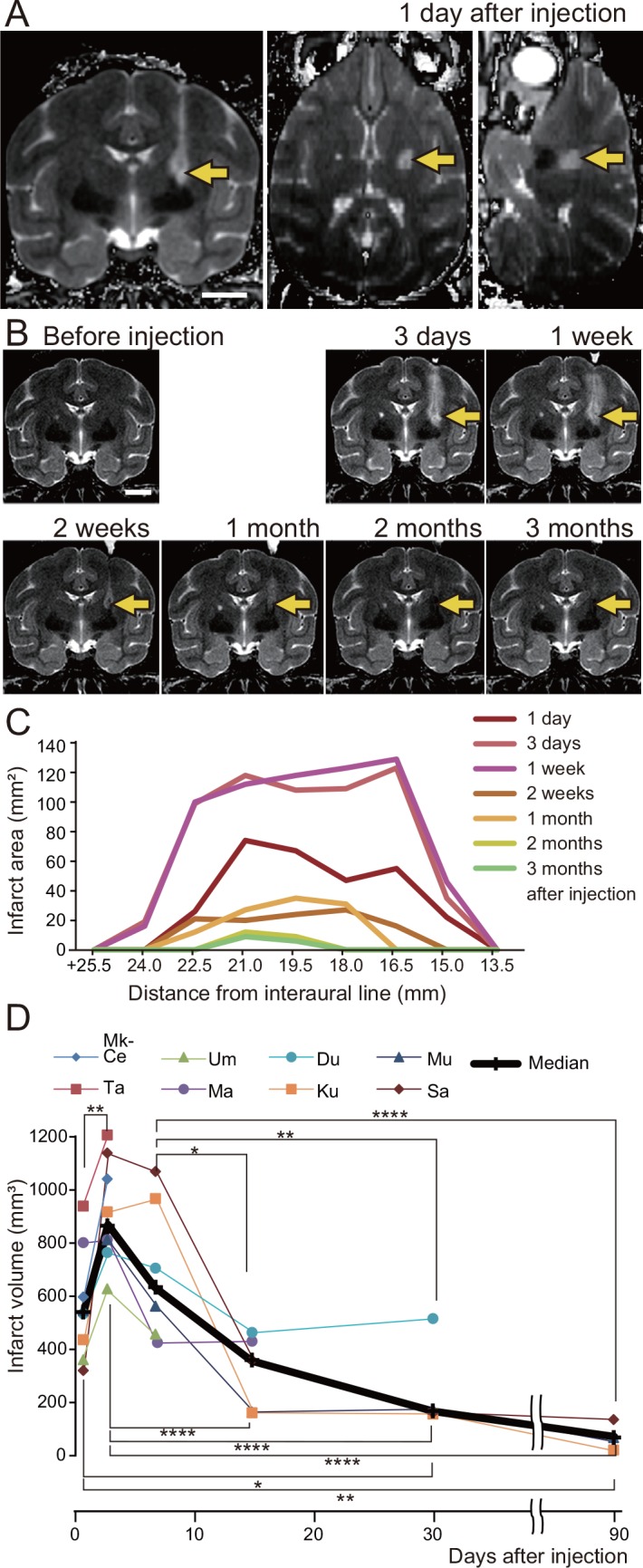
Infarct after endothelin-1 (ET-1) injection. (A, B) T2-weighted MR images of a macaque’s brain (Mk-Ku), showing the location of the infarct 1 day after ET-1 injection (A, coronal, axial, and sagittal images), and the temporal changes in the infarct during the 3 months after ET-1 injection (B, coronal images). The coronal images are taken 19.5 mm rostral to the interaural line. Arrows indicate the infarct. Scale bars = 10 mm. (C) Temporal changes in infarct expansion in successive coronal images are shown as a function of rostral distance from the interaural line. In this macaque (Mk-Ku), 19.5 and 21 mm rostral to the interaural line correspond to the levels at which motor tracts from the hand area of the primary motor cortex descend. (D) Temporal changes in infarct volume, as calculated for all ET-1 injected macaques (macaque identification numbers are given in the key). Kruskal-Wallis one-way analysis of variance indicated a significant difference of the median infarct volume among the periods after ET-1 injection (P < 0.0001), and Dunn’s post-hoc tests revealed that the infarct volumes at 3 days and 1 week after injection were higher than those in the remaining periods (*P < 0.05, **P < 0.01, ****P < 0.0001).

To evaluate spatiotemporal changes in the infarcts, MRI scans were performed before ET-1 injection and 1 day, 3 days, 1 week, 2 weeks, 1 month, and 3 months after injection. The MRI protocols were the same as described above. The area of infarct was seen as a hypointense infarct core with a surrounding hyperintense rim on T2-weighted MR images, as in previous studies [26–29]. The unbiased volumes of the infarct were calculated on the basis of Cavalieri’s principle [30] by using StereoInvestigator imaging software (MBF Bioscience, Williston, VT). The tracks of the injection needles were often visible as hyperintensities in T2-weighted images (Fig 1A). No hyperintensity was observed in the macaque used in a pilot study in which the needle was inserted, but no ET-1 was injected to confirm accurate localization of the needle tip in the posterior internal capsule (data not shown). Therefore, the hyperintense tracks were thought to result from the backflow of ET-1, although we attempted to minimize the backflow of ET-1 through the needle tracks, as described above. We included the hyperintense tracks in the infarct volume measures.

In addition, changes in the neuronal structure in layer V of M1 were evaluated by histological analysis. Tissue preparation was performed as previously described [31]. The macaque monkeys were deeply anesthetized with sodium pentobarbital (35–50 mg/kg, *i*.*v*.) and then perfused through the ascending aorta with 0.5 L of ice-cold saline containing sodium heparin (1000 units/mL), followed by ice-cold fixative consisting of 4% paraformaldehyde and 0.1% glutaraldehyde in phosphate buffer (pH 7.4). The volume of fixative perfused was 1 L/kg body weight. After perfusion, coronal sections (16 μm thickness) were made at the level of the superior genu of the central sulcus, as in our previous studies [3, 20, 32, 33]. We measured the size of the neuronal cell bodies in layer V of M1 by using a procedure similar to that used in our previous studies [3, 32, 33]. Briefly, the Nissl-stained sections were digitized by using StereoInvestigator imaging software (MBF Bioscience). We then measured the size of 50 neuronal cell bodies from layer V of the forelimb area of M1, within 3 mm of the central sulcus on coronal sections, by using Image J software (National Institutes of Health, Bethesda, MD). The measurements were performed by a person blinded to the treatment of the macaques.

### Statistical Analyses

Statistical significance was assessed by nonparametric tests including the Mann-Whitney U-test and Kruskal–Wallis one-way analysis of variance followed by Dunn’s post-hoc test.

## Results

The area of infarct 1 day after injection included the posterior internal capsule, where the motor tracts from the hand area of M1 descend (arrows in Fig 1A; *i*.*e*., the white matter lateral to the caudate nucleus/thalamus and medial to the putamen/globus pallidus on coronal sections from the rostrocaudal level, which includes the rostral end of the central sulcus to that, which includes the superior genu of the central sulcus) [24, 25]. The median and interquartile range of the percentage of infarct volume within the internal capsule was 82.3 [79.6–83.8] (Table 1). The infarct expanded between 3 days and 1 week; it then decreased until the end of the behavioral experiment at 3 months after ET-1 injection (Fig 1B, 1C and 1D).

**Fig 2:**
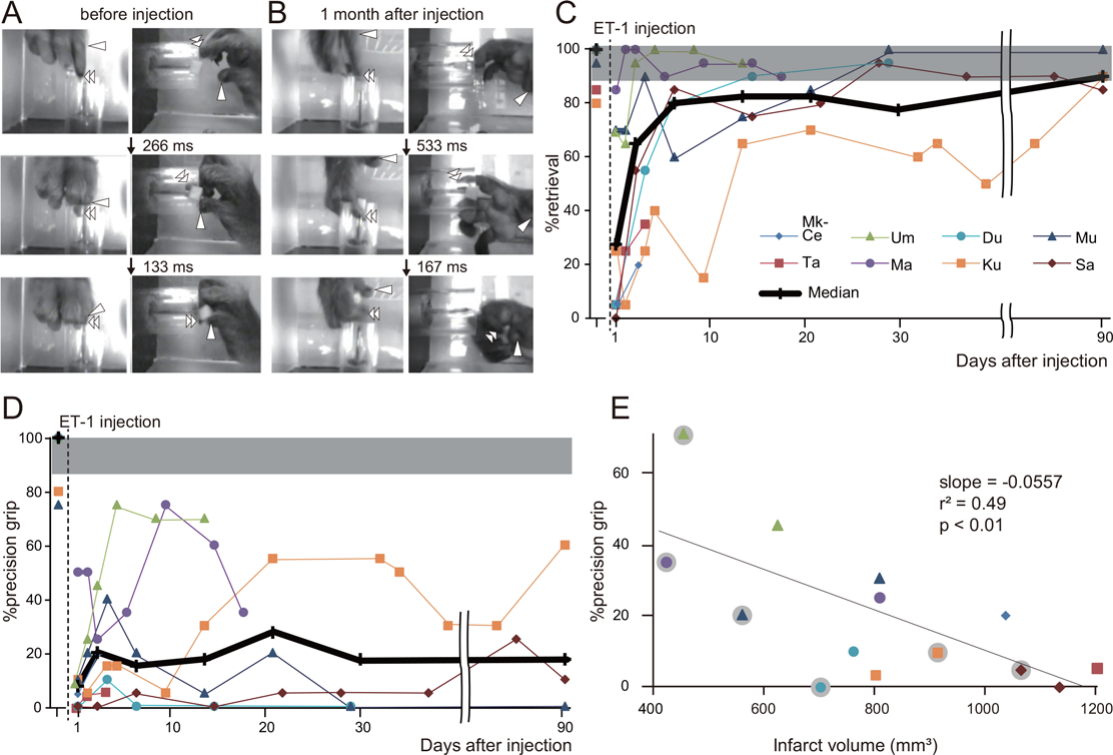
Behavioral changes after endothelin-1 (ET-1) injection. (A, B) Sequence of photographs showing hand and digit movements of a macaque (Mk-Du) performing the small-object retrieval task before (A) and at 1 month after (B) ET-1 injection. Upper, middle, and lower rows show both top and side views at the moments when the macaque inserts the index finger through the aperture of the cylindrical tube; when the tip of the index finger contacts the food morsel; and when the macaque pulls the index finger out of the tube, respectively. Arrowhead, thumb tip; double arrowheads, index finger tip. Before ET-1 injection, use of the precision grip involving the tips of both the index finger and the thumb was observed in almost all trials. At 1 month after injection, the macaque raked out the food morsel with the index finger and then held it in the proximal joints of the thumb. (C, D) Time course of changes in the percentage of successful retrieval (C) and precision grip (D) in all of ET-1-injected macaques. Shaded gray region represents the 95% confidence interval for performance before injection. The number of successful retrievals progressively increased over the first week; by 3 months after injection, it had reached the 95% confidence level. The median value for precision grip increased gradually in the first 3 weeks after injection; it then virtually plateaued and at 3 months was still below the 95% confidence level. (E) Correlation between infarct volume and successful achievement of precision grip 3 days or 1 week after ET-1 injection. Each symbol represents data from one macaque; symbols without gray circles are data for 3 days after injection, whereas symbols with gray circles are those for 1 week after injection. The success rate in achieving a precision grip at 3 days or 1 week was negatively correlated with the infarct volume.

Before ET-1 injection, all animals performed the small-object retrieval task smoothly using a precision grip, holding the morsel between the tips of the index finger and thumb (Fig 2A). Immediately after ET-1 injection, impairment of motor function in the contralateral forelimb was observed. There was no apparent impairment of movement of other body parts, such as the face or hindlimb, and the macaques showed no apparent difficulty in eating, drinking, or moving. The macaques showed impaired performance in the small-object retrieval task; the rates of successful retrieval and precision grip the day after injection were below the 95% confidence level for performance before injection (Fig 2C and 2D). The median percentage of successful retrievals in the small-object retrieval task increased rapidly over the week after injection (Fig 2C). It increased gradually thereafter and by 3 months was within the 95% confidence interval for performance before the injection. In contrast, the percentage of precision grip was below the 95% confidence level at 3 months after injection (Fig 2D).

After the injection, the macaques frequently retrieved the morsel using an alternative grip strategy because gross hand movements recovered whereas impairment of dexterous movements, such as independent digit movements, remained. Typically, the macaques raked out the morsel with the index finder and held it in the proximal joints of the thumb (Fig 2B). The infarct volume of each macaque was negatively correlated with precision grip performance 3 days and 1 week after injection (*P* < 0.01, linear regression analysis; Fig 2E), suggesting that individual differences in infarct volume may have accounted for the differences in hand performance. In contrast, no correlation between infarct volume and precision grip performance was observed in later periods (*P* > 0.05, data not shown).

**Fig 3:**
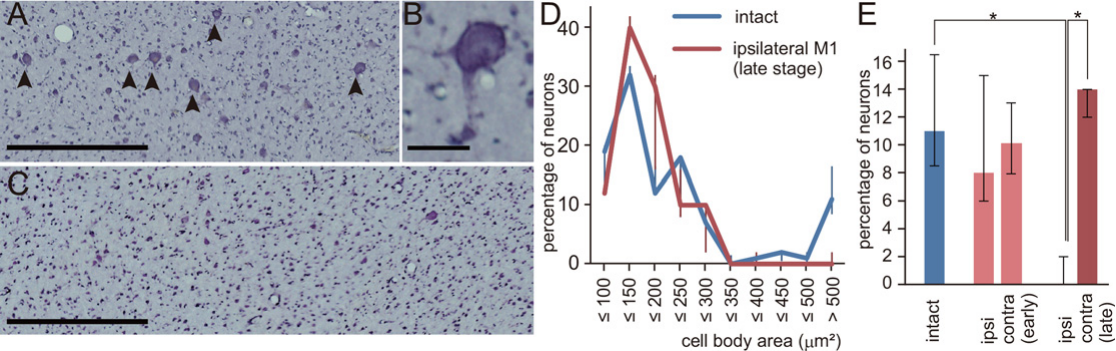
Histological changes after endothelin-1 (ET-1) injection. (A-C) Nissl-stained sections of layer V in the hand area of the primary motor area (M1) in a normal intact macaque (A, B) and a macaque 1 month after ET-1 injection (C, Mk-Du). Large pyramidal neurons (>30 μm in diameter) are distributed in the intact M1 (arrowheads in A), as shown in the higher-magnification photomicrograph (B). In contrast, large pyramidal neurons are rarely observed in M1 of the ET-1-injected macaque (C). Scale bars in (A, C) = 500 μm, and scale bar in (B) = 50 μm. (D) Percentage of neurons in each size range in the hand area of M1. Median and interquartile ranges are shown for M1 in intact macaques (n = 6) and for the ipsilateral M1 in ET-1 injected macaques with survival periods of 3 weeks or more (n = 5). (E) Median percentages and interquartile ranges of large pyramidal neurons (area >500 μm^2^) in the intact M1 (n = 6) and in the ipsilateral and contralateral M1 in the early (≤2 weeks, n = 3) and late (≥ 3 weeks, n = 5) stages after injection. The percentage in the ipsilateral M1 in the late stage was significantly lower than those in the contralateral and intact M1 (*P < 0.05, Kruskal–Wallis one-way analysis of variance and Dunn’s post-hoc test).

As a first step in investigating changes in brain structure induced by ET-1 injection, we measured the size of neurons in layer V of M1. In the intact M1, there was an abundance of large neurons (>30 μm in diameter, Fig 3A and 3B), from which the descending motor tracts originate [34–36]. Quantitative analysis of the percentage of neurons in each size range showed that there was a certain percentage of large neurons with a cell body area of > 500 μm^2^ in the hand area of the intact M1 (Fig 3D). In contrast, at 3 weeks after injection and beyond, there were fewer large neurons in layer V of M1 ipsilateral to the ET-1 injection site (Fig 3C). The percentage of neuronal cell bodies larger than 500 μm^2^ was significantly lower than that in the intact M1 and on the contralateral side (*P* < 0.05, Kruskal-Wallis one-way analysis of variance and Dunn’s *post hoc* test; Fig 3D and 3E). No significant change in neuronal size was observed in the contralateral M1 or in the ipsilateral M1 2 weeks or earlier after ET-1 injection (Fig 3E).

## Discussion

Here, we induced focal infarcts in the posterior internal capsule of macaques, resulting in impaired hand movements. Because a negative correlation between infarct volume and precision grip performance was observed at both 3 days and 1 week after injection, the degree of infarct expansion may have been a cause of the impairment in hand movements during the early stage. Precision grip impairment remained several months after ET-1 injection, when the infarct volume had decreased, suggesting that irreversible changes in brain structure were present in the late stage after injection.

Histological analysis showed that retrograde atrophy or degeneration of the motor projection neurons in M1 was a candidate for these irreversible changes. This atrophy was observed only during the late stage (3 weeks and later), suggesting that Wallerian degeneration and subsequent atrophy of M1 neurons occurred over several weeks after ET-1 injection. Although atrophy or degeneration has also been observed in the M1 neurons of macaques after creation of a lesion in the lateral corticospinal tract (l-CST) at the cervical spinal cord [33, 37], it was less severe than that in the present ET-1-injected macaques: the percentage of neuronal cell bodies larger than 500 μm^2^ was 6.6% in the macaques 3 months after l-CST lesion [33], whereas the median percentage was 0 in the ET-1-injected macaques. This difference may reflect the possibility that neurons subjected to insult close to the cell body are more susceptible to damage than those subjected to insult far from the cell body [38]. The present model is thus appropriate for investigating selective neuronal loss that occurs in preserved tissue after stroke [39].

We previously compared the motor recovery of macaques that had received intensive motor training with those that received no training after the creation of M1 lesions, and showed that the recovery of precision grip could be promoted by post-lesion training [1]. The behavioral changes in the present ET-1-injected macaques were similar to those of M1-lesioned macaques without post-lesion training, in that gross hand movements recovered whereas dexterous movements, including precision grip, did not recover to preinjection levels. A study of the effects of post-lesion rehabilitative training in ET-1-injected monkeys is an important next step.

Finally, we should point out that the present ET-1 injection model does not exactly reproduce the human pathophysiology of lacunar infarction. Although the majority of the lacunar infarcts in human patients are caused by occlusion of a single penetrating vessel, ET-1 injection caused several microvessels to be constricted simultaneously [40–43]. Additionally, ET-1 is localized not only to vascular endothelial cells but also to neurons and astrocytes in the brain [44–47], and the receptors for ET-1 are expressed in activated astrocytes and microglia as well as vascular endothelial cells [48, 49]. Thus, ET-1 may also modulate the functions of neurons and glia. Because of these limitations, the present ET-1 injection model is not suitable for investigating the acute phase of infarction, but is better suited for studying the progression of neurological damage and the recovery of function that occurs during chronic stages after white matter infarcts in primates. A recent internal capsule stroke model in the rat and common marmoset showed long-lasting motor deficits that persisted for at least 1 month and 10 days, respectively [12, 17]. The present study followed the macaques for 3 months after ET-1 injection and confirmed that impairment of dexterous hand movements remained until the end of the behavioral and imaging experiments at 3 months. Thus, the present model may have an advantage over other experimental models in testing therapeutic interventions that last for several months, such as rehabilitative training, after white matter infarcts.

## Supporting Information

S1 ARRIVE Guidelines Checklist We followed the ARRIVE (Animal Research: Reporting of *In Vivo* Experiments) guidelines and the ARRIVE Checklist is available. (PDF)

## References

[1] Murata Y, Higo N, Oishi T, Yamashita A, Matsuda K, Hayashi M, et al Effects of motor training on the recovery of manual dexterity after primary motor cortex lesion in macaque monkeys. Journal of neurophysiology. 2008;99(2):773–86. Epub 2007/12/21. 10.1152/jn.01001.2007 . 18094104

[2] Murata Y, Higo N, Hayashi T, Nishimura Y, Sugiyama Y, Oishi T, et al Temporal plasticity involved in recovery from manual dexterity deficit after motor cortex lesion in macaque monkeys. The Journal of neuroscience: the official journal of the Society for Neuroscience. 2015;35(1):84–95. Epub 2015/01/09. 10.1523/JNEUROSCI.1737-14.2015 25568105 PMC4287160

[3] Murata Y, Higo N, Oishi T, Isa T. Increased expression of the growth-associated protein-43 gene after primary motor cortex lesion in macaque monkeys. Neuroscience research. 2015;98:64–9. Epub 2015/05/12. 10.1016/j.neures.2015.04.007 . 25959053

[4] Vilensky JA, Gilman S. Lesion of the precentral gyrus in nonhuman primates: a pre-medline bibliography. Int J Primatology. 2002;23(6):1319–33.

[5] Nudo RJ. Mechanisms for recovery of motor function following cortical damage. Current opinion in neurobiology. 2006;16(6):638–44. Epub 2006/11/07. 10.1016/j.conb.2006.10.004 . 17084614

[6] Darling WG, Pizzimenti MA, Morecraft RJ. Functional recovery following motor cortex lesions in non-human primates: experimental implications for human stroke patients. Journal of integrative neuroscience. 2011;10(3):353–84. Epub 2011/10/01. 10.1142/S0219635211002737 21960307 PMC3689229

[7] Higo N. Effects of rehabilitative training on recovery of hand motor function: a review of animal studies. Neuroscience research. 2014;78:9–15. Epub 2013/10/02. 10.1016/j.neures.2013.09.008 . 24080147

[8] Friel KM, Nudo RJ. Recovery of motor function after focal cortical injury in primates: compensatory movement patterns used during rehabilitative training. Somatosensory & motor research. 1998;15(3):173–89. Epub 1999/01/05. . 9874517 10.1080/08990229870745

[9] Schiemanck SK, Kwakkel G, Post MW, Kappelle LJ, Prevo AJ. Impact of internal capsule lesions on outcome of motor hand function at one year post-stroke. Journal of rehabilitation medicine. 2008;40(2):96–101. Epub 2008/05/30. 10.2340/16501977-0130 . 18509572

[10] Wenzelburger R, Kopper F, Frenzel A, Stolze H, Klebe S, Brossmann A, et al Hand coordination following capsular stroke. Brain: a journal of neurology. 2005;128(Pt 1):64–74. Epub 2004/10/09. 10.1093/brain/awh317 . 15471902

[11] Rosso C, Colliot O, Valabregue R, Crozier S, Dormont D, Lehericy S, et al Tissue at risk in the deep middle cerebral artery territory is critical to stroke outcome. Neuroradiology. 2011;53(10):763–71. Epub 2011/07/27. 10.1007/s00234-011-0916-5 . 21789602

[12] Blasi F, Whalen MJ, Ayata C. Lasting pure-motor deficits after focal posterior internal capsule white-matter infarcts in rats. Journal of cerebral blood flow and metabolism: official journal of the International Society of Cerebral Blood Flow and Metabolism. 2015;35(6):977–84. Epub 2015/02/05. 10.1038/jcbfm.2015.7 . 25649992 PMC4640262

[13] Puentes S, Kurachi M, Shibasaki K, Naruse M, Yoshimoto Y, Mikuni M, et al Brain microvascular endothelial cell transplantation ameliorates ischemic white matter damage. Brain research. 2012;1469:43–53. Epub 2012/07/10. 10.1016/j.brainres.2012.06.042 . 22771710

[14] Frost SB, Barbay S, Mumert ML, Stowe AM, Nudo RJ. An animal model of capsular infarct: endothelin-1 injections in the rat. Behavioural brain research. 2006;169(2):206–11. Epub 2006/02/25. 10.1016/j.bbr.2006.01.014 . 16497394

[15] Lecrux C, McCabe C, Weir CJ, Gallagher L, Mullin J, Touzani O, et al Effects of magnesium treatment in a model of internal capsule lesion in spontaneously hypertensive rats. Stroke; a journal of cerebral circulation. 2008;39(2):448–54. Epub 2008/01/05. 10.1161/STROKEAHA.107.492934 . 18174487

[16] Tanaka Y, Imai H, Konno K, Miyagishima T, Kubota C, Puentes S, et al Experimental model of lacunar infarction in the gyrencephalic brain of the miniature pig: neurological assessment and histological, immunohistochemical, and physiological evaluation of dynamic corticospinal tract deformation. Stroke; a journal of cerebral circulation. 2008;39(1):205–12. Epub 2007/12/01. 10.1161/STROKEAHA.107.489906 . 18048856

[17] Puentes S, Kaido T, Hanakawa T, Ichinohe N, Otsuki T, Seki K. Internal capsule stroke in the common marmoset. Neuroscience. 2015;284:400–11. Epub 2014/12/03. 10.1016/j.neuroscience.2014.10.015 . 25453768

[18] Courtine G, Bunge MB, Fawcett JW, Grossman RG, Kaas JH, Lemon R, et al Can experiments in nonhuman primates expedite the translation of treatments for spinal cord injury in humans? Nature medicine. 2007;13(5):561–6. . 17479102 10.1038/nm1595 PMC3245971

[19] Yamamoto T, Oishi T, Higo N, Murayama S, Sato A, Takashima I, et al Differential expression of secreted phosphoprotein 1 in the motor cortex among primate species and during postnatal development and functional recovery. PloS one. 2013;8(5):e65701 Epub 2013/06/07. 10.1371/journal.pone.0065701 23741508 PMC3669139

[20] Higo N, Sato A, Yamamoto T, Nishimura Y, Oishi T, Murata Y, et al SPP1 is expressed in corticospinal neurons of the macaque sensorimotor cortex. The Journal of comparative neurology. 2010;518(13):2633–44. Epub 2010/05/27. 10.1002/cne.22356 . 20503431

[21] Kaas JH. The evolution of the complex sensory and motor systems of the human brain. Brain research bulletin. 2008;75(2–4):384–90. Epub 2008/03/12. 10.1016/j.brainresbull.2007.10.009 18331903 PMC2349093

[22] Yanagisawa M, Kurihara H, Kimura S, Tomobe Y, Kobayashi M, Mitsui Y, et al A novel potent vasoconstrictor peptide produced by vascular endothelial cells. Nature. 1988;332(6163):411–5. Epub 1988/03/31. 10.1038/332411a0 . 2451132

[23] Weatherall D. The use of non-human primates in research. Working group report. Available: https://royalsociety.org/~/media/Royal_Society_Content/policy/publications/2006/Weatherall-Report.pdf. Accessed 2006.

[24] Schmahmann JD, Pandya DN. Fiber Pathways of the Brain. New York, NY: Oxford University Press; 2009. 654 p.

[25] Morecraft RJ, Herrick JL, Stilwell-Morecraft KS, Louie JL, Schroeder CM, Ottenbacher JG, et al Localization of arm representation in the corona radiata and internal capsule in the non-human primate. Brain: a journal of neurology. 2002;125(Pt 1):176–98. Epub 2002/02/09. . 11834603 10.1093/brain/awf011

[26] Zhao B, Shang G, Chen J, Geng X, Ye X, Xu G, et al A more consistent intraluminal rhesus monkey model of ischemic stroke. Neural regeneration research. 2014;9(23):2087–94. Epub 2015/02/07. 10.4103/1673-5374.147936 25657726 PMC4316474

[27] Kleinschnitz C, Schutz A, Nolte I, Horn T, Frank M, Solymosi L, et al In vivo detection of developing vessel occlusion in photothrombotic ischemic brain lesions in the rat by iron particle enhanced MRI. Journal of cerebral blood flow and metabolism: official journal of the International Society of Cerebral Blood Flow and Metabolism. 2005;25(11):1548–55. Epub 2005/05/27. 10.1038/sj.jcbfm.9600151 . 15917747

[28] West GA, Golshani KJ, Doyle KP, Lessov NS, Hobbs TR, Kohama SG, et al A new model of cortical stroke in the rhesus macaque. Journal of cerebral blood flow and metabolism: official journal of the International Society of Cerebral Blood Flow and Metabolism. 2009;29(6):1175–86. Epub 2009/04/23. 10.1038/jcbfm.2009.43 19384334 PMC2828874

[29] Mack WJ, Komotar RJ, Mocco J, Coon AL, Hoh DJ, King RG, et al Serial magnetic resonance imaging in experimental primate stroke: validation of MRI for pre-clinical cerebroprotective trials. Neurological research. 2003;25(8):846–52. Epub 2003/12/13. 10.1179/016164103771953943 . 14669528

[30] Mayhew TM. A review of recent advances in stereology for quantifying neural structure. J Neurocytol. 1992;21(5):313–28. . 1607876 10.1007/BF01191700

[31] Higo N, Oishi T, Yamashita A, Matsuda K, Hayashi M. Cell type- and region-specific expression of protein kinase C-substrate mRNAs in the cerebellum of the macaque monkey. The Journal of comparative neurology. 2003;467(2):135–49. Epub 2003/11/05. 10.1002/cne.10850 . 14595765

[32] Higo N, Oishi T, Yamashita A, Murata Y, Matsuda K, Hayashi M. Expression of protein kinase-C substrate mRNA in the motor cortex of adult and infant macaque monkeys. Brain research. 2007;1171:30–41. Epub 2007/09/01. 10.1016/j.brainres.2007.07.054 . 17761152

[33] Higo N, Nishimura Y, Murata Y, Oishi T, Yoshino-Saito K, Takahashi M, et al Increased expression of the growth-associated protein 43 gene in the sensorimotor cortex of the macaque monkey after lesioning the lateral corticospinal tract. The Journal of comparative neurology. 2009;516(6):493–506. Epub 2009/08/13. 10.1002/cne.22121 . 19672995

[34] Murray EA, Coulter JD. Organization of corticospinal neurons in the monkey. The Journal of comparative neurology. 1981;195(2):339–65. . 7251930 10.1002/cne.901950212

[35] Toyoshima K, Sakai H. Exact cortical extent of the origin of the corticospinal tract (CST) and the quantitative contribution to the CST in different cytoarchitectonic areas. A study with horseradish peroxidase in the monkey. Journal fur Hirnforschung. 1982;23(3):257–69. . 7130676

[36] Jones EG, Wise SP. Size, laminar and columnar distribution of efferent cells in the sensory-motor cortex of monkeys. The Journal of comparative neurology. 1977;175(4):391–437. 410849 10.1002/cne.901750403

[37] Wannier T, Schmidlin E, Bloch J, Rouiller EM. A unilateral section of the corticospinal tract at cervical level in primate does not lead to measurable cell loss in motor cortex. Journal of neurotrauma. 2005;22(6):703–17. Epub 2005/06/09. 10.1089/neu.2005.22.703 . 15941378

[38] Fu SY, Gordon T. The cellular and molecular basis of peripheral nerve regeneration. Molecular neurobiology. 1997;14(1–2):67–116. Epub 1997/02/01. 10.1007/BF02740621 . 9170101

[39] Baron JC, Yamauchi H, Fujioka M, Endres M. Selective neuronal loss in ischemic stroke and cerebrovascular disease. Journal of cerebral blood flow and metabolism: official journal of the International Society of Cerebral Blood Flow and Metabolism. 2014;34(1):2–18. Epub 2013/11/07. 10.1038/jcbfm.2013.188 24192635 PMC3887360

[40] Bailey EL, McCulloch J, Sudlow C, Wardlaw JM. Potential animal models of lacunar stroke: a systematic review. Stroke; a journal of cerebral circulation. 2009;40(6):e451–8. Epub 2009/04/25. 10.1161/STROKEAHA.108.528430 . 19390080

[41] Micheli S, Corea F. Lacunar versus non-lacunar syndromes. Frontiers of neurology and neuroscience. 2012;30:94–8. Epub 2012/03/02. 10.1159/000333426 . 22377873

[42] Arboix A, Marti-Vilalta JL. Lacunar stroke. Expert review of neurotherapeutics. 2009;9(2):179–96. Epub 2009/02/13. 10.1586/14737175.9.2.179 . 19210194

[43] Fisher CM. Lacunes: Small, Deep Cerebral Infarcts. Neurology. 1965;15:774–84. Epub 1965/08/01. . 14315302 10.1212/wnl.15.8.774

[44] Fuxe K, Tinner B, Staines W, Hemsen A, Hersh L, Lundberg JM. Demonstration and nature of endothelin-3-like immunoreactivity in somatostatin and choline acetyltransferase-immunoreactive nerve cells of the neostriatum of the rat. Neuroscience letters. 1991;123(1):107–11. Epub 1991/02/11. . 1676496 10.1016/0304-3940(91)90169-t

[45] MacCumber MW, Ross CA, Snyder SH. Endothelin in brain: receptors, mitogenesis, and biosynthesis in glial cells. Proceedings of the National Academy of Sciences of the United States of America. 1990;87(6):2359–63. Epub 1990/03/01. 2156267 10.1073/pnas.87.6.2359 PMC53686

[46] Yoshimoto S, Ishizaki Y, Kurihara H, Sasaki T, Yoshizumi M, Yanagisawa M, et al Cerebral microvessel endothelium is producing endothelin. Brain research. 1990;508(2):283–5. Epub 1990/02/05. . 2407312 10.1016/0006-8993(90)90407-3

[47] Naidoo V, Naidoo S, Mahabeer R, Raidoo DM. Cellular distribution of the endothelin system in the human brain. Journal of chemical neuroanatomy. 2004;27(2):87–98. Epub 2004/05/04. 10.1016/j.jchemneu.2003.12.002 . 15121213

[48] Gadea A, Schinelli S, Gallo V. Endothelin-1 regulates astrocyte proliferation and reactive gliosis via a JNK/c-Jun signaling pathway. The Journal of neuroscience: the official journal of the Society for Neuroscience. 2008;28(10):2394–408. Epub 2008/03/07. 10.1523/JNEUROSCI.5652-07.2008 18322086 PMC2695974

[49] Loo LS, Ng YK, Zhu YZ, Lee HS, Wong PT. Cortical expression of endothelin receptor subtypes A and B following middle cerebral artery occlusion in rats. Neuroscience. 2002;112(4):993–1000. Epub 2002/06/29. . 12088756 10.1016/s0306-4522(02)00043-x

